# Combining Radiation Therapy with ALK Inhibitors in Anaplastic Lymphoma Kinase-Positive Non-Small Cell Lung Cancer (NSCLC): A Clinical and Preclinical Overview

**DOI:** 10.3390/cancers13102394

**Published:** 2021-05-15

**Authors:** Delphine Antoni, Hélène Burckel, Georges Noel

**Affiliations:** 1 Paul Strauss Comprehensive Cancer Center, Radiobiology Laboratory, Institut de Cancérologie Strasbourg Europe (ICANS), Strasbourg University, UNICANCER, 67000 Strasbourg, France; h.burckel@icans.eu (H.B.); g.noel@icans.eu (G.N.); 2 Department of Radiotherapy, ICANS, Institut de Cancérologie Strasbourg Europe, 17 rue Albert Calmette, CEDEX, 67200 Strasbourg, France

**Keywords:** non-small cell lung cancer, ALK, tyrosine kinase inhibitors, radiotherapy

## Abstract

**Simple Summary:**

The use of tyrosine kinase inhibitors has significantly improved the outcome of patients with ALK (anaplastic lymphoma kinase)-rearranged non-small cell lung cancer. Combining drugs with radiotherapy could increase its effectiveness on tumors or overcome resistance to ALK-targeted therapies. In this review, we provide a clinical and preclinical overview of combining radiation therapy with ALK inhibitors in anaplastic lymphoma kinase-positive non-small cell lung cancer, and we also propose several approaches to overcome resistance to ALK-targeted therapies with a combination of radiation and tyrosine kinase inhibitors.

**Abstract:**

Over the past years, the identification of genetic alterations in oncogenic drivers in non-small cell lung cancer (NSCLC) has significantly and favorably transformed the outcome of patients who can benefit from targeted therapies such as tyrosine kinase inhibitors. Among these genetic alterations, anaplastic lymphoma kinase (ALK) rearrangements were discovered in 2007 and are present in 3–5% of patients with NSCLC. In addition, radiotherapy remains one of the cornerstones of NSCLC treatment. Moreover, improvements in the field of radiotherapy with the use of hypofractionated or ablative stereotactic radiotherapy have led to a better outcome for localized or oligometastatic NSCLC. To date, the effects of the combination of ALK inhibitors and radiotherapy are unclear in terms of safety and efficacy but could potently improve treatment. In this manuscript, we provide a clinical and preclinical overview of combining radiation therapy with ALK inhibitors in anaplastic lymphoma kinase-positive non-small cell lung cancer.

## 1. Introduction

The management of non-small cell lung cancer (NSCLC) has become challenging in recent years with the development and efficacy of new therapies such as immunotherapy and the identification of genetic alterations in oncogenic drivers susceptible to targeted therapies, such as tyrosine kinase inhibitors (TKIs). The major characterized molecular targets in NSCLC are epidermal growth factor receptor (EGFR) and anaplastic lymphoma kinase (ALK). Several randomized phase III trials have demonstrated improved efficiency using EGFR or ALK inhibitors with regard to cytotoxic chemotherapies such as platinum agents combined with pemetrexed or docetaxel, indicating that targeted therapies should be the standard care for some selected patients [1,2,3,4]. In addition, improvements in the field of radiotherapy have led to better outcomes for localized, advanced or oligometastatic NSCLC, with the use of intensity-modulated radiation therapy (IMRT), hypofractionated or ablative stereotactic radiotherapy (SBRT) and image-guided and adaptive radiotherapy [5,6,7,8]. These local treatments were shown to be effective in primitive tumors as well as in oligometastatic disease and are currently delivered as normofractionated or hypofractionated therapy [9,10].

To date, the use of combined treatments of TKIs and radiotherapy in terms of toxicity and efficacy is still unclear, especially for ALK inhibitors. In clinical practice, a precautionary principle is applied, withholding TKI treatment during radiotherapy. However, this concomitant strategy may improve the efficacy of radiotherapy through potential synergistic effects, as well as the combination of chemotherapy and radiotherapy.

**Figure 1:**
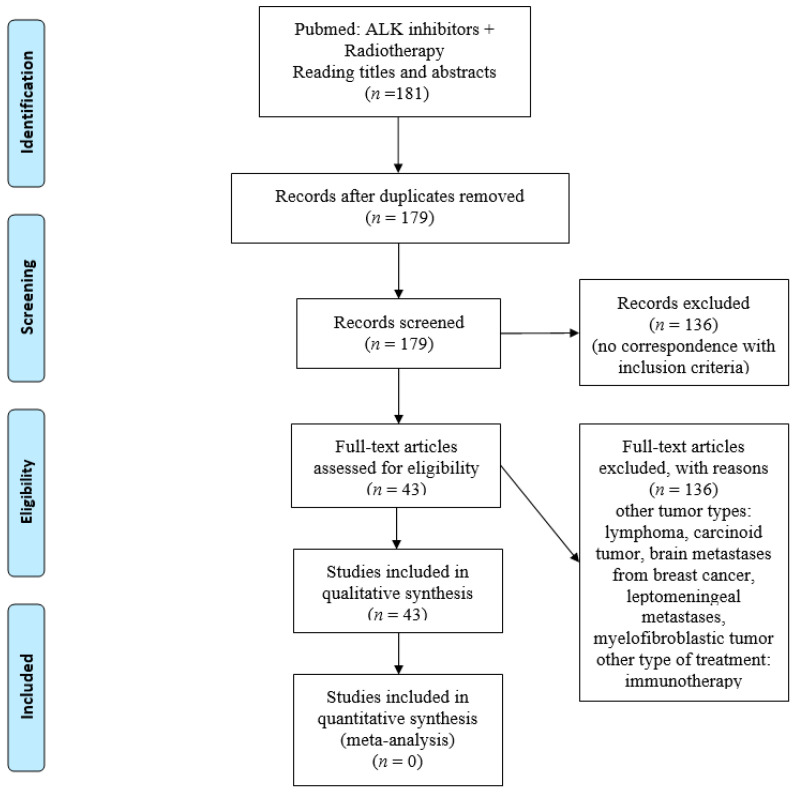
Flow chart of published selected articles in the PubMed database search.

For a review of the effects of concomitant radiotherapy and ALK TKI inhibitors in ALK-positive NSCLC, an article search followed the “PRISMA method” [11]. Articles corresponding to the terms “ALK inhibitors” and “radiotherapy” were searched in the PubMed database. Studies published between 2012 and January 2021 in English were included. Only studies about NSCLC were collected for the analysis. Overall, 181 articles were retrieved. Among them, 136 articles were excluded because they did not meet the inclusion criteria. Ultimately, 43 studies were included (Figure 1). This review provides a clinical and preclinical overview of studies examining combinations of ALK-targeted therapies and radiotherapy used sequentially or concomitantly in patients with ALK-positive NSCLC.

## 2. ALK Inhibitors

Discovered in 2007, ALK rearrangements are present in 3–5% of patients with NSCLC [12,13]. The echinoderm microtubule-associated protein-like 4-anaplastic lymphoma kinase (EML4-ALK) fusion gene, resulting from an inversion within chromosome 2p, is frequently and mutually exclusive with KRAS and EGFR mutations. ALK rearrangements are significantly correlated with PI3K (phosphatidylinositol 3-kinase)/AKT/mTOR (mammalian target of rapamycin) and RAS (rat sarcoma)/RAF/MEK (mitogen-activated protein kinase or Erk kinase) signaling pathways, leading to cell survival, proliferation and growth differentiation and translation [14]. In this molecular subgroup of NSCLC, ALK rearrangements critically confer sensitivity to ALK inhibition. Patients with ALK rearrangements are younger than those without ALK rearrangements, and most patients have little or no exposure to tobacco and present predominantly adenocarcinomas [12].

### 2.1. Crizotinib

Crizotinib, a first-in-class ALK/ROS1/MET inhibitor, has been evaluated in two randomized phase III trials (PROFILE 1014 and 1007). Compared to first- and second-line cytotoxic chemotherapeutic drugs, crizotinib significantly improved the overall response rate, progression-free survival (PFS) and quality of life [7,8,15]. In the cohort study of Kwak et al., 82 patients with advanced ALK-positive disease were treated with crizotinib. After a mean treatment duration of 6.4 months, 57% of the patients showed confirmed partial or complete responses, and 33% had stable disease. Crizotinib was approved initially by the FDA (Food and Drug Administration) in 2011 for the treatment of advanced ALK-positive NSCLC. Despite the efficacy of targeted therapies in NSCLC, the development of resistance is common and represents a major clinical challenge. In the case of progression after first-line targeted therapy, the molecular mechanisms of resistance to targeted therapy should be identified by investigating drug resistance mutations using circulating tumor DNA and/or rebiopsy. Resistance to targeted therapies can be classified as either intrinsic or acquired [16]. Indeed, from preclinical data, the sensitivity profiles of the different ALK inhibitors are different depending on the drug resistance mutations considered [17].

Gainor et al. analyzed 103 rebiopsies from ALK-positive patients progressing on various ALK inhibitors [17]. A majority of these ALK-positive NSCLC patients had acquired resistance to ALK inhibitors. The frequency and spectrum of ALK resistance mutations were different according to the ALK inhibitors used for treatment. In cases with progression after crizotinib, the percentage of ALK mutations was 20%. In 51 ALK-positive patients progressing on crizotinib, the most common ALK resistance mutation was the gatekeeper mutation L1196M, which is analogous to EGFR, T790M and G1269A. Other ALK resistance mutations included C1156Y, G1202R, I1171T, S1206Y and E1210K. These resistance mutations can be divided into two main categories: on the one hand, mutations which interest the residues involved in the direct contact with the inhibitor, with the consequence to compromise its binding due to steric hindrance. On the other hand, mutations of the residues distant from the inhibitor-binding site have been identified, and this second class of mutations promote conformational changes increasing ALK kinase activity [18,19]. A few data have been established for some of them. For example, L1196M significantly increases the catalytic efficiency of processing ATP using nonphosphorylated ALK [20], whereas I1171T confers a potent gain of function on the Alk protein as indicated by ligand-independent autophosphorylation [21]. G1202R has been demonstrated to confer drug resistance also in the context of ALK fusions, but has not been biochemically characterized and therefore, its effect on Alk protein function is unknown [22].

### 2.2. ALK Inhibitors of the Second and Third Generation

Alectinib is a second-generation ALK inhibitor and has demonstrated increased efficiency to crizotinib in terms of 12-month overall survival without events (68.4 vs. 48.7%) (hazard ratio (HR) for death or progression of 0.47 (95% CI 0.34–0.65), *p* < 0.001) [23]. Recently, updated global survival data have been reported, with a 4-year survival rate of 64.5% (95% CI 55.6–73.4) in the alectinib arm and 52.2% (95% CI 42.6–64.8) in the crizotinib arm [24]. The effectiveness of this drug is particularly demonstrated by the control of brain metastases or the time until the onset of brain metastases [25,26,27,28]. Alectinib should be considered the standard of care in first-line ALK-positive advanced NSCLC, as it was safer and more effective than crizotinib as a first-line chemotherapeutic treatment for patients with ALK-rearranged lung adenocarcinoma [29] (Table 1). Additionally, alectinib showed successful management of ALK-rearranged lung squamous cell carcinoma [30]. Moreover, the ALTA-1L study compared brigatinib (a second-generation ALK inhibitor) with crizotinib. Patients were ALK inhibitor-naive but may have received chemotherapy. The HR for PFS was 0.49 (95% CI 0.33–0.74, *p* < 0.001), with a median survival of 9.8 months in the crizotinib arm and not reached in the brigatinib arm (Table 1). The 1-year PFS rates were 67% (95% CI 56–75) in the brigatinib arm and 43% (95% CI 32–53) in the crizotinib arm. Survival without cranial progression was higher in the brigatinib group (HR = 0.27 (95% CI 0.13–0.54)) than the crizotinib group [31]. Alectinib and brigatinib have received FDA approval and should be considered the standard of care in first-line advanced ALK-positive NSCLC [32]. In the absence of comparative data, the use of brigatinib compared to alectinib remains to be specified.

Similarly, the frequency and type of mutation according to the type of ALK inhibitor was previously investigated. Thus, ALK resistance mutations are more common with brigatinib (71%), ceritinib (54%) and alectinib (53%), while they remain rare after crizotinib (20%). The G1202R mutation is the most common (preclinical sensitivity to crizotinib and alectinib) and is observed in 43% of cases after brigatinib, 29% after alectinib and 21% after ceritinib [17].

In the case of second-generation ALK inhibitor resistance, the presence of ALK resistance mutations is highly predictive of sensitivity to lorlatinib, a third-generation ALK inhibitor. Notably, lorlatinib was the only ALK inhibitor to potently inhibit ALK phosphorylation in cases with all single ALK secondary mutations, including G1202R. The frequency of this mutation increased significantly after treatment with second-generation TKIs. Moreover, cell lines without ALK resistance mutations after failure of second-generation ALK inhibitors acquire resistance to lorlatinib, which may indicate loss of ALK dependency.

Lorlatinib, a third-generation ATP-competitive selective ALK and ROS1 inhibitor, was specifically designed for drug resistance mutation sites and optimized to penetrate the blood–brain barrier, and has been approved as a second- or third-line treatment [34]. Lorlatinib was compared to crizotinib in the phase III CROWN study. An intermediate analysis showed that the median PFS was 9.3 months with crizotinib, whereas it was not reached with lorlatinib (HR = 0.28; 95% CI 0.19–0.41) (Table 1). To date, lorlatinib has not been approved as a first-line treatment in advanced ALK-positive NSCLC in France [33]. The different tyrosine kinase inhibitors and their indications are summarized in Table 2.

Due to these analyses, Gainor et al. could define an “ALKogramme” describing the efficacy of the ALK inhibitors crizotinib, ceritinib, alectinib, brigatinib and lorlatinib by their absolute cellular ALK phosphorylation levels and their IC50 value, according to 15 different mutation statuses [17]. Moreover, it should be noted that in some cases, several types of ALK resistance mutations coexist; in the evaluation published by Gainor et al., six specimens were identified with more than two ALK resistance mutations [17].

In the case of progression to an accessible local treatment area, the realization of this local treatment must be considered with the continuation of ALK inhibitors. If there is a slow and asymptomatic progression, ALK inhibitor continuation is proposed but with early reassessment. In the case of initial progression under ALK inhibitors, treatment adaptation to the available molecular resistance profile is recommended. In the absence of molecular data, alectinib is suggested if not previously used or brigatinib, ceritinib or lorlatinib [35,36]. The same principles are followed for subsequent progressions [37,38].

### 2.3. ALK Variants

The heterogeneous responses to ALK inhibitors have led to the identification of several ALK variants. Chromosomal inversion within the short arm of chromosome 2 does not always occur in the same location. EML4-ALK fusion has more than 21 different variants according to the location of the fusion. Variant 1, variant 3a/b and variant 2 are the most common variants and are found in 33, 29 and 10% of cases, respectively [39,40,41]. Variant 1 corresponds to the fusion of exon 13 of EML4 to exon 20 of ALK (E13; A20); variant 3a/b consists of the fusion of exon 6a or 6b of EML4 to exon 20 of ALK (E6a/b; A20), whereas variant 2 is the fusion of exon 20 of EML4 and to exon 20 of ALK (E20; A20). In vitro, Heuckmann et al. presented a difference in sensitivity to crizotinib in Ba/F3 cell line models affected by different variants. Variant 2 was significantly more sensitive to ALK inhibitors than variants 3b and 1 [42]. In a retrospective study, Yoshida et al. evaluated the efficacy of crizotinib in 55 patients presenting tumors with ALK variants [43]. Variants 1, 2, 3a/3b and miscellaneous were found in 54, 14, 13 and 20% of cases, respectively. The objective response rate was not significantly higher for patients with variant 1 than for other patients (74 vs. 63%) (*p* = 0.72). Consequently, the median PFS time was significantly longer in patients with variant 1 than in those without variant 1 (11 vs. 4.2 months, respectively (*p* < 0.05)). Thus, ALK variant status might affect the efficacy of ALK inhibitors; therefore, the precise ALK genotype should be detected and taken into account for the management of NSCLC patients.

### 2.4. Other Bypass Signaling Pathways

Although ALK resistance mutations appear to be the predominant mechanism of resistance, alternative mechanisms of resistance must exist since no ALK mutation was found in 44% of rebiopsies. Targeted NGS was performed on 27 biopsy specimens post-ceritinib, post-alectinib or post-brigatinib treatment, and genetic alterations were common in at least one nonrelated ALK gene, including TP53. Various bypass signaling pathways, such as ligand-mediated HER2/3 activation and protein kinase C activation (via P2Y receptors), cKIT amplification, insulin-like growth factor 1 receptor (IGF-1R) activation and upregulation of the expression of SRC proto-oncogenes and nonreceptor tyrosine kinases, have been identified as drivers of crizotinib resistance [44,45,46]. Moreover, preclinical studies have suggested that epithelial–mesenchymal transition (EMT), such as loss of E-cadherin and gain of vimentin expression, is associated with resistance to crizotinib and ceritinib [16,47]. Other off-target mechanisms of resistance, especially to second-generation ALK inhibitors, including MAPK pathway reactivation, SRC activation, PIK3CA mutations and MET amplification, have been identified [46,47,48]. Unlike crizotinib, second-generation ALK inhibitors do not have anti-MET activity.

## 3. Radiotherapy of NSCLC

Used alone, before or after surgery, or concomitantly with systemic treatments, radiotherapy is an essential therapeutic option in NSCLC [49]. Many improvements have been achieved in recent years in the field of radiotherapy in lung cancer. IMRT has resulted in a higher conformation and a better sparing of organs at risk compared to 3D-conformal radiotherapy. Likewise, respiratory-gated radiotherapy allows us to limit the impact of respiratory movements during irradiation and to reduce dosimetric parameters predictive of pulmonary, cardiac and esophageal acute and late toxicities. Image-guided radiotherapy and adaptive radiotherapy increase the precision in delivering irradiation. Commonly, curative normofractionated radiotherapy in lung cancer is delivered in daily doses of 1.8–2.2 Gy for 5–8 weeks in the range of 54 to 66 Gy [9]. Normofractionated radiotherapy is mostly used in cases of locally advanced lung cancer associated with chemotherapy or as a neoadjuvant or adjuvant treatment. However, the effectiveness of radiotherapy is often limited by the intrinsic resistance of cancer cells and radiation-induced toxicity in nearby healthy tissues. The SBRT technique in NSCLC, for which good efficacy and safety are observed in clinical practice due to many sophisticated advances in RT technologies, has become essential. Delivering higher doses of radiation per fraction and thus increasing the biologically effective dose (BED) in a shorter time appears to be a safe option. For ALK-positive NSCLC, hypofractionated and ablative radiotherapy is one proposable strategy, since many patients with oncogene-driven NSCLC treated with ALK inhibitors experience limited sites of disease progression (oligoprogressive disease) accessible to local treatment without modification of beneficial targeted agents and discontinuation of ALK inhibitors. Local ablative therapy of cranial or extracranial oligoprogressive disease safely prolongs the disease control obtained with ALK inhibitors [50,51].

Investigating the effects of the combined treatment of ALK inhibitors with radiotherapy is particularly important in terms of efficacy and safety. On the one hand, it is necessary to ensure the safety and acceptable serious adverse events of concurrent ALK inhibitor treatment plus cranial or extracranial radiotherapy so that the ALK inhibitor will not be interrupted for the duration of radiotherapy and therefore not risk a loss of efficacy of the ALK inhibitor. On the other hand, if ALK inhibitors could increase radiosensitivity and/or if radiotherapy could reduce ALK-TKI resistance, this knowledge will allow us to adapt the schedule and sequence of administration of the ALK inhibitor and radiotherapy fractionation, especially in cases of SBRT where the number of fractions is low, ranging from one to eight.

## 4. Combined Treatment of Radiotherapy and ALK Inhibitors: A Clinical Overview

The combination of radiation treatment and systemic therapies is a relevant approach to improve the outcome in several kinds of tumors, such as head and neck tumors or gynecologic cancers [52]. Indeed, several factors related to cancer radiation sensitivity can be promising targets for radiosensitizers: redistribution in the cell cycle or the capacity to repair radiation-induced DNA damage remain the most evaluated [53,54]. ALK-positive NSCLC presents specific metastatic patterns, especially compared to EGFR-positive or EGFR/ALK wild-type advanced NSCLC, with significantly more distant nodal metastasis [55], which in some cases could benefit from radiation therapy. Target-based therapies combined with radiotherapy could be some alternatives to chemotherapy but deserve to be more deeply studied. However, to date, only one ongoing trial is examining unresectable locally advanced NSCLC harboring ALK rearrangement. Moreover, the randomized phase II trial RTOG 1306 (NCT01822496) is now over and assessed nine ALK-positive patients treated with crizotinib for 12 weeks, followed by chemotherapy with cisplatin/etoposide or paclitaxel/carboplatin plus concurrent radiotherapy in one arm; in the other arm, seven patients received a combination of radiotherapy and chemotherapy only [56]. The median PFS was 14.7 months for the patients in the crizotinib arm and was not reached in the standard group. The complete and partial response rates were 66.7 and 75%, respectively. The median distant PFSs were 20.1 months and not reached. No patient presented any grade 3–5 adverse events in the two groups. This study stopped accrual early due to unmet target accrual goals, with 59 subjects recruited out of 234 planned.

Approximately 40% of NSCLC patients develop brain metastases during the course of their disease. This rate reaches 60% in ALK-positive patients treated with crizotinib [57]. This progression results from a blood–brain barrier (BBB) pharmacokinetic issue rather than true biological resistance. Currently, stereotactic radiotherapy is the cornerstone of the treatment for brain metastases, allowing the shielding of radiation-sensitive brain structures. However, in some cases, whole brain radiation therapy (WBRT) is required, involving a risk of cognitive function deterioration. As the outcome of ALK-positive patients is fairly good, the preservation of cognition and quality of life is a relevant goal. Consequently, postponing WBRT by using ALK inhibitors that cross the BBB is a suitable option. In a recent study, Yang et al. evaluated various therapies in 76 patients presenting ALK fusions who developed brain metastases [58]. The median OS of patients treated with crizotinib was not reached compared to the 10-month median OS for patients who were irradiated or received non-ALK inhibitor treatment (*p* = 0.012 and *p* < 0.001, respectively). Notably, for the 19 patients who received crizotinib and cranial RT, the 3-year OS rate was 70%. ALK inhibitors were safely associated with WBRT, but their combination with radiosurgery remains to be evaluated [59,60].

Local SBRT is an effective option to overcome resistance to ALK inhibitors in progressive sites without discontinuation. Gan et al. evaluated SBRT in oligoprogressive disease while maintaining ALK inhibitor treatment. Thirty patients were enrolled in the study, and 14 presented disease progression in four or fewer extracranial sites accessible to local therapy (SBRT, conformal RT or surgery). For patients treated with SBRT, the 6- and 12-month local control rates were 100 and 86%, respectively. The duration of crizotinib treatment was longer for patients who received SBRT than for those who did not, with 28 vs. 10 months, respectively. No severe radiation-induced toxicity was observed [50]. Borghetti et al. investigated the role of conventional radiotherapy and SBRT in 50 patients with ALK metastatic NSCLC. Radiotherapy was delivered before, after or concomitant with TKIs in 16, 18 and 66% of the patients, respectively. The TKI was never suspended due to an adverse event, and only two grade 3 toxicities were reported. The median durations of TKI therapy were 14.2, 9.7 and 8.3 months when TKI was delivered concomitant with, before or after RT, respectively. A significant benefit in OS was observed in the SBRT group compared to the conventional RT group (*p* = 0.043) [61]. According to the limited data available about the safety of combined SBRT and TKI, the sequential combination seems to be well tolerated since two retrospective studies did not describe any acute or late grade 3–5 toxicities. However, in most of the trials or reports, TKI was never delivered on days of RT [51,62].

Gomez et al. performed a multi-institutional randomized phase II study of local consolidative therapy versus maintenance therapy or observation for patients with oligometastatic NSCLC without progression after front-line systemic therapy. Among the 49 patients enrolled in the study, only two presented ALK-positive lung cancer and were treated with crizotinib. Toxicity was similar in the two groups, and PFS was improved in the local therapy group vs. the maintenance group, with 11.9 vs. 3.9 months, respectively [63].

## 5. Combined Treatment with Radiotherapy and ALK Inhibitors: A Preclinical Overview

The effects of combining ALK inhibitors and ionizing radiation on the downstream signaling pathways that regulate the proliferation or survival of EML4-ALK-positive lung cancer cells remain to be established. The use of ALK inhibitors as potential novel radiosensitizing agents in NSCLC has been studied in vitro and in vivo, but some aspects remain unclear. The first-generation ALK inhibitor crizotinib has mostly been studied. Sun et al. examined the effect of crizotinib alone (0.4 μmol/L) and in combination with radiation on downstream signaling, apoptosis and radiosensitivity in two NSCLC cell lines, H3122 harboring an EML4-ALK fusion and H460 without this mutation [64]. The H3122 cells treated with crizotinib for two hours followed by 6 Gy radiation displayed an increased level of cellular apoptosis compared to the cells that were only irradiated. Moreover, a clonogenic assay was performed, and the H3122 cells exposed to crizotinib showed greater radiosensitivity (dose enhancement ratio (DER) = 1.43; *p* < 0.0001). This result was confirmed in the H2228 cell line, which was also ALK-positive (DER 1.23; *p* < 0.008). Tumati et al. evaluated the combination of crizotinib and radiation in vitro in five human NSCLC cell lines with varying expression levels of c-MET and EML4-ALK: A549, H460, H3122, H2228 and H1993. Crizotinib induced a MET inhibitor effect [65]. H3122 and H2228 harbor an EML4-ALK fusion, H1993 and H2228 have significant MET gene amplification and overexpress constitutively phosphorylated MET receptors and A549 and H460 exhibit neither the EML4-ALK fusion nor MET gene amplification. No radiosensitization was shown in this panel of NSCLC cell lines, but the combination with irradiation was roughly additive. In a study by Dai et al., the addition of crizotinib (200 nM) significantly reduced cell proliferation compared to irradiation alone in ALK-positive H3122 cells [66]. Combined therapy was even less efficient in inhibiting A549 cell growth than radiotherapy alone, suggesting a potential radioprotective effect of an ALK inhibitor on this cell line. Clonogenic survival confirmed a significant radiosensitizing effect of crizotinib (100 nM) in ALK-positive H3122 cells with photons and carbon ions (sensitizer enhancement ratio (SER) = 1.39 (*p* < 0.05) and SER 1.29 (*p* < 0.05), respectively). However, crizotinib only modestly sensitized A549 cells with photons (SER = 1.08 (*p* ≤ 0.05)), whereas no significant difference was observed with carbon ions. Crizotinib (100 nM) exerted proapoptotic effects on H3122 cells (27%), and these effects were increased by radiotherapy delivered with photons, with 60% observed with the combined treatments.

Dai et al. evaluated a second-generation ALK inhibitor, TAE684, in the same cell lines and under the same conditions [67]. TAE684 is a small-molecule inhibitor of ALK activity that has not been approved in clinical practice. However, in vitro, TAE684 (40 nM) markedly overcame crizotinib resistance in the H3122 cell line, decreasing cell growth, suppressing ALK phosphorylation and inducing apoptosis [68]. In vivo, TAE684 alone also showed impressive antitumor activity against H3122 xenograft tumors. Dai et al. demonstrated a synergistic effect of the combination of TAE684 and photon irradiation in H3122 cells. A significant 56% reduction in cell proliferation was observed compared to that of radiation alone (*p* < 0.01) [67]. Clonogenic survival assays demonstrated a significant radiosensitizing effect of TAE684 (SER = 1.6 (*p* < 0.01)). Moreover, irradiation delivered after TAE684 led to a 3-fold enhancement of apoptotic activity compared to that of TAE684 monotherapy (*p* < 0.05). Investigations were also performed with carbon ions and confirmed a significant radiosensitive effect in ALK-positive H3122 cells with TAE684 (SER = 1.61, *p* < 0.05). Recently, Fleschutz et al. compared the effects of three ALK inhibitors from different generations: crizotinib, ceritinib and alectinib in A549 (wild-type) and EML4-ALK fusion A549 isogenic cell lines. Cells were treated with ALK inhibitors (1 μM for all ALK inhibitors) followed by irradiation delivered at a dose of 6 or 10 Gy with photons [69]. The 185IG cell line was more sensitive to crizotinib than to ceritinib and alectinib administered as monotherapy. In the 185IG cell line, alectinib plus irradiation at 10 Gy led to the most significant reduction in survival compared to irradiation alone, whereas the combination of ceritinib and irradiation showed the best synergistic effect. However, combined treatments led to similar levels of DNA double-strand breaks (DSBs) in both cell lines compared with irradiation alone. Finally, these investigations of Fleschutz et al. resulted in no specific effect on ALK-mutated cell lines treated with irradiation alone or combined treatments, regardless of the ALK inhibitor used.

Classically, the cell cycle arrest induced by systemic treatment is an important factor in improving the efficacy of radiotherapy through a radiosensitizing effect. Radioresistance varies in different phases of the cell cycle. Cancer cells are more radiosensitive in the late G1 phase than the G2-M phase of the cell cycle [54]. The radiation resistance increases during the S phase and is maximal at the end of the S phase. Radioresistance in the S phase is related to elevated levels of DNA synthesis and repair enzymes. In response to ionizing radiation, cell cycle blockade in the G1 or G-/M phase of the cell cycle is observed. Cell cycle blockade in the G1 phase of the cell cycle allows time for DNA damage recognition and repair, whereas during G2-M phases of the cell cycle, there is less time for adequate repair, explaining increased radiosensitivity. It has been shown that anti-EGFR and radiation induce the accumulation of tumor cells in G1 and G2-M phases, respectively, with a reduction in cells in S-phase. The combination of TKIs with irradiation led to a further reduction in cells in S-phase, enhancing apoptosis and inhibition of EGFR autophosphorylation and of RAD51, and all these effects together led to an increase in radiosensitivity [70,71]. G1/S arrest in H3122 cells resulting in apoptosis was obtained with ceritinib [72] or with WY-135, which is an ALK/ROS1 dual inhibitor [73]. In contrast, when alectinib, ceritinib or crizotinib was combined with irradiation, G2 cell cycle arrest rates were comparable to or lower than those observed with radiation alone [69]. In vivo, in H3122 xenograft models, combined treatment led to less tumor proliferation than either treatment alone. These observations might be secondary to a greater inhibition of microvascular density and perfusion [64,65,66]. In contrast, in vivo, Tumati et al. did not observe any significant enhancement of tumor growth delay in response to crizotinib and irradiation [65]. To date, radiosensitization with ALK inhibitors in EML4-ALK NSCLC remains unclear.

## 6. Combined Radiotherapy and ALK Inhibitors: An Approach to Overcome Resistance to ALK-Targeted Therapies?

Identifying resistance to ALK-targeted therapies has led to the development of novel next-generation inhibitors, rational combined therapeutics and the increased use of local ablative therapies, especially radiation therapy.

### 6.1. Cooperative Effects of Radiotherapy and ALK Inhibitors

The results of combinational chemoradiotherapy can be divided into three categories: i. Supra-additivity (synergism or radiosensitization): the addition of a drug to radiation treatment increases radiation-induced cell death by more than the sum of the two treatment modalities used separately. ii. Additivity: cell death upon the addition of a drug to radiation treatment equals the sum of the two treatment modalities used separately. iii. Infra-additivity (antagonism or radioprotection): the addition of a drug protects the cells from radiation. If the drug shows greater protection for normal cells than tumor cells, this property is also desirable. The aim of combined chemoradiotherapy is to increase the therapeutic index, which can be achieved by either a decrease in the effective dose or an increase in the toxic dose [74]. Different types of drug radiotherapy interactions or radiobiological cooperation are proposed when combining radiotherapy and systemic agents, especially novel targeted drugs such as ALK inhibitors: spatial cooperation (different therapeutic approaches target different anatomical sites of the tumor), biological cooperation (use of different mechanisms to target distinct cell populations to increase local tumor control), temporal cooperation or modulation (cell kinetics cooperation: redistribution to more radiosensitive phases, reoxygenation, repopulation after radiation and cells repair), cytotoxic enhancement (achieved by molecular interaction between the two treatment modalities; for example, if the target is DNA, the objective is to increase the number of single or double-strand breaks) and selective protection of normal cells (by addition of selective radiation protectors to normal cells prior to or during radiation therapy) [75].

ALK fusion proteins are involved in multiple cellular signaling pathways with oncogenic potential: Ras/extracellular signal-regulated kinase (Erk), phosphatidylinositol 3-kinase (PI3K)/Akt and Janus protein tyrosine kinase (JAK)/STAT. These pathways are involved in cell cycle progression, survival, proliferation and angiogenesis [76]. In particular, PI3K/Akt activation enhances a cascade of responses, which have consequences for survival, proliferation and cell growth, as well as DNA double-strand break repair and tumor angiogenesis through hypoxia-inducible factor 1α (HIF-1α) and vascular endothelial growth factor [77]. There are few studies on the molecular mechanisms underlying the possible cooperative effects of radiotherapy and ALK inhibitors. However, the majority of DNA double-strand breaks caused by irradiation are repaired by nonhomologous end-joining (NHEJ) [78]. Considering EGFR, Toulany et al. showed that EGFR/PI3K/Akt signaling activates DNA-dependent protein kinase (DNA-PKcs), regulating DNA double-strand break repair by NHEJ [79]. Das et al. showed that in the case of decreased DNA-PKcs, the EGFR-mediated radioprotective effect is lost [80].

### 6.2. Effect of Hypoxia

Kogita et al. studied the effect of hypoxia in response to two ALK inhibitors, first- and second-generation crizotinib and alectinib, in the H3122 cell line [47]. The sensitivity of the H3122 cell line to ALK inhibitors was evaluated under normoxic and hypoxic conditions. This cell line was shown to be resistant to ALK inhibitors during a hypoxic state. This observation could be explained by the fact that hypoxia induced EMT, which is associated with resistance to TKIs of the first and second generations, as previously stated. To overcome this resistance due to hypoxia, researchers could use a combination with HIF inhibitors since HIF1A knockdown, which promoted MET, abolished the resistance [81,82,83].

**Figure 2:**
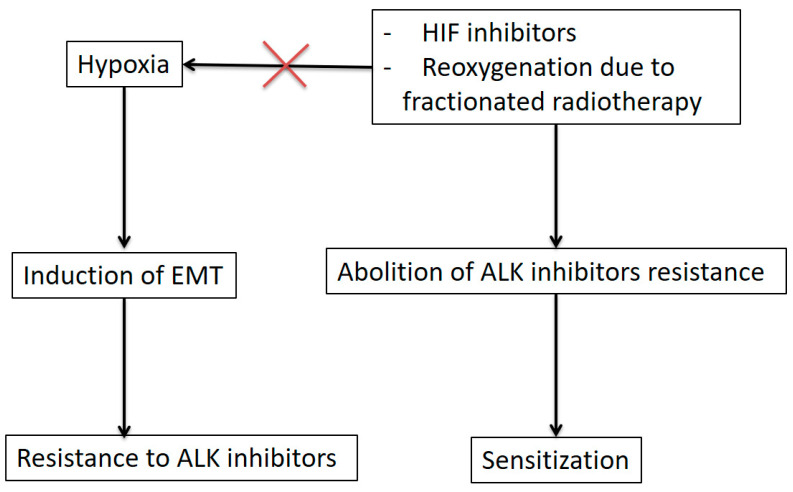
Role of hypoxia in ALK inhibitor resistance and the ability of radiation therapy to overcome this resistance. ALK: anaplastic lymphoma kinase, EMT: epithelial–mesenchymal transition, HIF: hypoxia inducible factor.

Early radiobiological studies found that the main mechanisms of action of radiation were related to DNA damage and subsequent cell death. However, the radiobiology of ablative radiation is still being investigated. Indeed, the radiobiological principles of 5R have yet to be determined. Notably, the roles of hypoxia and vascularization, more specifically, angiogenesis and vasculogenesis, seem to be dominant. Ionizing radiation will be more harmful to better oxygenated cells [84]. According to the principle of reoxygenation, tumors may be acutely or chronically hypoxic. This oxygenation status may change during treatment. Within a tumor, there is a population of chronically hypoxic cells due to the poor vasculature of tumors resistant to radiation. Additionally, some cells present acute hypoxia due to transient closure of vessels, while other cells are well oxygenated. Oxygen is a powerful radiosensitizer because it can amplify the effects of free radicals, increasing the indirect effects of ionizing radiation on DNA. Irradiation fractionation leads to the death of oxygenated cells close to the vessels, allowing an increase in the available oxygen; the most distant hypoxic cells will then be able to be reoxygenated between each fraction, and their radiosensitivity will be increased. As reoxygenation occurs between fractions, hypoxic cells may acquire some radiosensitivity. Therefore, reoxygenation of acutely hypoxic cells between daily fractions is an important aspect of fractionated radiotherapy. Concurrent fractionated radiotherapy and TKIs should be preferred to radiosurgery to increase the number of cells that will have an adequate supply of oxygen. The role of hypoxia in resistance to ALK inhibitors and the role of radiation therapy in overcoming this resistance are summarized in Figure 2.

### 6.3. Role of c-MYC

Other identified bypass signaling pathways could lead to relevant approaches to overcome resistance to ALK inhibitors. Pilling et al. described the MYC signaling pathway in resistance to crizotinib [85]. Through a genome-wide RNAi-based synthetic lethal screen in H2228 and H3122 ALK-positive cell lines, c-MYC binding protein (MYCBP) was identified as a determinant of crizotinib sensitivity. Further investigations have shown that treatment with ALK inhibitors reduced MYC and MYCBP transcript levels in all ALK-positive cell lines in a dose-dependent manner. ALK was able to regulate the transcriptional expression of MYC and activate c-MYC transactivation of c-MYC target genes. Consequently, inhibition of c-MYC increased crizotinib sensitivity. Thus, ALK enhanced the MYC signaling axis, and its inhibition might be a target to prevent or overcome resistance. Rihawi et al. also suggested that MYC overexpression in ALK-positive NSCLC could be a potential mechanism associated with resistance to ALK inhibitors [86].

The role of c-MYC in ALK inhibitor resistance has been identified in some tumor types (glioblastoma, neuroblastoma, etc.). Approximately 60% of glioblastomas express ALK, but ALK fusion proteins and mutations occur in only 1% [87]. Berberich et al. evaluated the effects of alectinib and irradiation in nonresistant and alectinib-resistant glioblastoma cells. In vitro, high expression of c-MYC and activation of the ERK1/2 pathway were associated with resistance against alectinib in ALK-expressing glioblastoma cells, and administration of a c-MYC inhibitor or MEK inhibitor was able to overcome TKI resistance and led to resensitization of resistant cells. Similar findings were observed in neuroblastoma driven by the expression of the ALK and MYCN genes with lorlatinib and crizotinib, respectively [88,89]. ALK amplification has also been reported in neuroblastoma almost invariably together with amplification of the adjacent gene MYCN, with possible synergistic effects in driving cell growth and survival [90].

Bonolo de Campos et al. evaluated 76 emerging FDA-approved oncology drugs in multiple myeloma and non-Hodgkin’s lymphoma cell lines [91]. It appeared that samples harboring a key oncogenic IRF4 (interferon regulatory factor 4) mutation were sensitive to the ALK inhibitors ceritinib and crizotinib. IRF4 is a lymphocyte-specific transcription factor for which a relevant target gene is c-MYC [92,93].

The MYC signaling pathway is also involved in the response to radiation. c-MYC may be linked to the DNA damage response pathway. Li et al. showed that c-MYC could play a role in regulating the repair of radiation-induced DSBs. Indeed, radiation can induce the formation of colocalized c-MYC and ƴ-H2AX foci in H1299 human non-small cell lung carcinoma cell lines derived from lymph nodes [94]. It has been shown that, in vitro and in vivo, c-MYC has an impact on the suppression of DSB repair, and V(D)J recombination may occur through inhibition of the nonhomologous end-joining pathway. Cui et al. investigated the function of c-MYC in the repair of DSBs induced by γ-ray irradiation in a c-MYC-silenced HeLa-630 cell line [95]. A reduction in DNA DSB repair was observed, and the repair kinetics of DSBs were delayed compared to those of the control HeLa-NC cells. The authors also demonstrated that depression of c-MYC expression largely attenuated the ionizing radiation-induced phosphorylation of ataxia telangiectasia mutated (ATM) and decreased the in vitro kinase activity of DNA-PKcs. The overexpression of c-MYC may account for the radioresistance of some tumor cell types. Consequently, these data suggest that inhibition of c-MYC expression could lead to increasing sensitivity to ALK inhibitors and radiation, because the MYC signaling pathway is involved in the response to ALK inhibitors and radiation.

### 6.4. Immunomodulatory Effects of Radiotherapy

**Figure 3:**
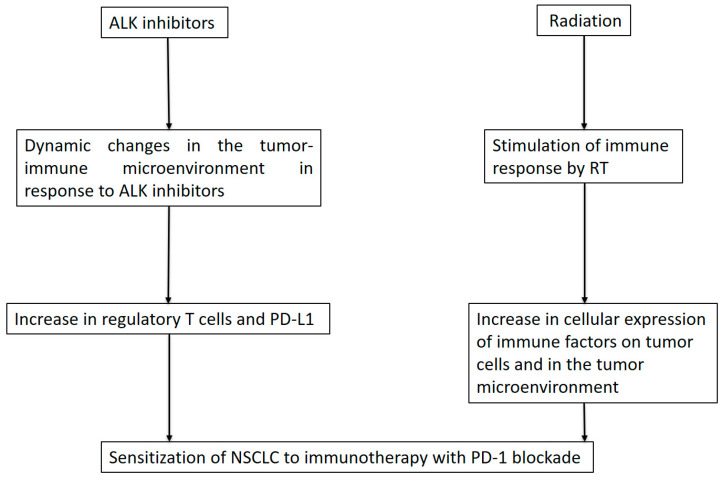
Role of the immune response to ALK inhibitors and role of radiation therapy. ALK: anaplastic lymphoma kinase, RT: radiotherapy, PD-L1: programmed death ligand 1, NSCLC: non-small cell lung carcinoma.

Immunotherapy with immune checkpoint inhibitors, specifically PD and PD-L1, has demonstrated efficiency in advanced NSCLC [96]. To date, immune checkpoint inhibitors have not shown efficacy in ALK-positive NSCLC. However, some dynamic changes in the tumor-immune microenvironment in response to ALK inhibitors, such as an increase in cells expressing regulatory T cells and programmed death-ligand 1 (PD-L1), have been observed in both the tumor and the stroma [97]. Lui et al. showed that the combination of crizotinib with cisplatin, which is known as nonimmunogenic cell death-inducing chemotherapy in NSCLC cells in vitro and in vivo, can lead to an increase in T lymphocyte infiltration and the expression of PD-1 and PD-L1. This phenomenon could lead to a strong sensitization of NSCLC to immunotherapy with PD-1 antibodies [98]. The combination sensitized NSCLC models to subsequent immunotherapy with PD-1 blockade, allowing the cure of more than 90% of established orthotopic cancers. The sequence of administration involving combined treatment with cisplatin and crizotinib, followed by PD-1 blockade one week later, showed that acute hepatotoxicity could be avoided [99]. These findings could be an interesting approach to overcome TKI resistance. Similarly, radiotherapy has immunomodulatory effects and not only cytotoxic effects on tumor cells, as it can stimulate the immune response through various mechanisms, inducing cellular expression of immune factors on both tumor cells and within the tumor microenvironment [100,101]. Immunogenic cell death is accompanied by exposure to tumor-associated antigens and damage-associated patterns that facilitate the uptake of tumor-associated antigens and their presentation to CD8+ tumor-specific cytotoxic T lymphocytes. Radiation can activate innate and adaptive immune systems and promote antitumor responses at irradiated sites but also at a distances from the metastatic sites, called the abscopal effect [102,103]. The role of immune effects in response to ALK inhibitors and radiotherapy and their effects in treatment by immunotherapy are summarized in Figure 3.

The different mechanisms of resistance to ALK inhibitors and the role of radiation therapy are summarized in Table 3.

## 7. Conclusions

The use of ALK inhibitors has significantly changed the management and outcome of patients with ALK-positive NSCLC. Patients can experience several ALK inhibitors of different generations during their disease as well as radiotherapy, especially in oligometastatic situations. The combination of radiotherapy and ALK inhibitors still needs to be evaluated in terms of safety and efficacy. However, radiotherapy could be an interesting approach to overcome resistance to ALK-targeted therapies.

## Figures and Tables

**Table 1:** Randomized studies evaluating first-generation tyrosine kinase inhibitors compared to second- or third-generation tyrosine kinase inhibitors in first-line treatment of metastatic non-small cell lung cancer with ALK rearrangement. TKI: tyrosine kinase inhibitor, PFS: progression-free survival, NE: not estimable, NR: not reached, HR: hazard ratio.

References	Study	TKI/Number of Patients	Median PFS (Months)	HR PFS	1 Year PFS (%)	1 Year Cerebral PFS (%)
Peters et al. 2017 [18] Mok et al. 2019 [24] Camidge et al. 2019 [28]	ALEX	Crizotinib (*n* = 151) Alectinib (*n* = 152)	10.9 (9.1–12.9) 34.8 (17.7–NE)	0.43 (0.32–0.58)	48.7 (40.4–56.9) 68.4 (61.0–75.9)	- -
Camidge et al. 2018 [31]	ALTA–1L	Crizotinib (*n* = 138) Brigatinib (*n* = 137)	9.8 (9.0–12.9) NR	0.49 (0.33–0.74) (*p* < 0.001)	43 (32–53) 67 (56–75)	61 (50–71) 78 (68–85)
Shaw et al. 2020 [33]	CROWN	Crizotinib (*n* = 147) Lorlatinib (*n* = 149)	9.3 (7.6–11.1) NR	0.28 (0.19–0.41) (*p* < 0.001)	39 (30–48) 78 (70–84)	60 (49–69) 96 (91–98)

**Table 2:** Indication of treatment of ALK-positive metastatic small-cell lung cancer. TKI: tyrosine kinase inhibitor, ALK: anaplastic lymphoma kinase, NTRK: neurotrophic receptor tyrosine kinase, Gen.: Generation.

TKI	Gen.	Posology	Target	FDA Approval for Treatment of ALK-Positive Metastatic NSCLC	Indication for Treatment of ALK-Positive Metastatic NSCLC
Alectinib	2	600 mg × 2/day	ALK	Yes	First-line
Brigatinib	2	90 mg × 1/day for 7 days then 180 mg × 1/day	ALK	Yes	First-line
Ceritinib	2	450 mg × 1/day	ALK	Yes	ALK TKI agent based on the resistance profile
Crizotinib	1	250 mg × 2/day	ALK ROS1	Yes	
Lorlatinib	3	100 mg × 1/day	ALK ROS1	Yes	ALK TKI agent based on the resistance profile

**Table 3:** Different mechanisms of resistance to ALK inhibitors and the role of radiation therapy. ALK: anaplastic lymphoma kinase, TKI: tyrosine kinase inhibitor.

Mechanisms of Resistance to ALK Inhibitors	Role of RADIOTHERAPY
ALK resistance mutations	In the case of slow progression under TKI
Other bypass signaling pathways (TP53, HER2/3 activation, protein kinase C activation, cKIT amplification, insulin-like growth factor 1-receptor activation, upregulation of SRC proto-oncogene expression, EMT, MAPK pathway reactivation, SRC activation, PIK3CA mutation, MET amplification)	In the case of slow progression under TKI
Role of hypoxia	Decreases resistance to TKIs
Inhibition of c-MYC	Improves sensitivity to TKIs
Role of immunomodulatory effects	Sensitizes to immunotherapy
